# Illuminating Fungal Infections with Bioluminescence

**DOI:** 10.1371/journal.ppat.1004179

**Published:** 2014-07-10

**Authors:** Nicolas Papon, Vincent Courdavault, Arnaud Lanoue, Marc Clastre, Matthias Brock

**Affiliations:** 1 Université François Rabelais de Tours, EA2106, Faculté de Pharmacie, Tours, France; 2 Microbial Biochemistry and Physiology, Leibniz Institute for Natural Product Research and Infection Biology, Hans Knoell Institute, Jena, Germany; The University of North Carolina at Chapel Hill, United States of America

## What Is Bioluminescence Imaging (BLI)?

**Figure 1:**
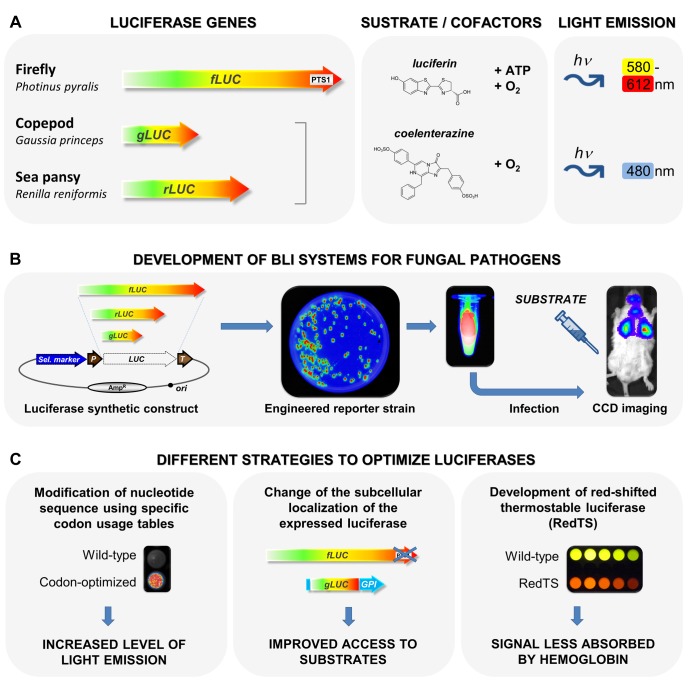
Development and optimization of bioreporters for fungal infection BLI. (**A**) The three luciferase-encoding genes used in *Aspergillus* and *Candida* species. The firefly (*Photinus pyralis*) *fLUC* gene encoding a luciferase (Fluc), which converts the substrate luciferin to oxy-luciferin in an ATP-dependent manner, the sea pansy (*Renilla reniformis*) *rLUC* and the copepod *Gaussia* (*Gaussia princeps*) *gLUC*, which both encode luciferases (Rluc and Gluc, respectively) that produce light emission from the substrate coelenterazine. All luciferases require oxygen for activity. (**B**) Schematic presentation of the different steps of the construction of fungal bioreporters and their use in BLI. A fungal strain is first genetically modified to stably express a luciferase gene as a reporter. Luciferase-expressing cells are used for animal infection and the substrate, luciferin or coelenterazine, is extemporaneously administered. Finally, light emitted from reporter cells is externally monitored in real time, using sensitive photon detectors based on cooled or intensified charge coupled devices (CCD). (**C**) Compilation of various strategies to optimize luciferases used in fungal infection BLI. This includes the adaptation of luciferase nucleotide sequences to the fungal host using specific codon usage tables (left panel), the change of the subcellular localization of the expressed luciferase either by removing (PTS1 of firefly luciferase) or by adding (GPI anchor for *Gaussia* luciferase) targeting sequences (middle panel), and the generation of red-shifted/thermostable firefly luciferases through enzyme redesign (right panel).

BLI is a powerful biophotonic imaging technology that allows in vivo visualization of temporal and spatial progression of infections in living organisms. BLI relies on the detection of visible light arising from an enzymatic reaction of oxidation known as bioluminescence. Originally, bioluminescence referred to the light emission of living organisms (e.g., bacteria, fungi, fish, insects, algae, and squid), which results from the oxidation of organic substrates mediated by catalysts named luciferases (Figure 1A) [1]. This fascinating natural phenomenon has been thus diverted to create bioluminescent microorganisms that are currently used in BLI as bioreporters. BLI has the advantages of being highly sensitive with excellent signal-to-noise ratios, and being non-invasive and nontoxic for animals. Such an approach has been applied in the past two decades to study the fate of tumor cells in various therapeutic settings and of several infectious agents including bacteria, viruses, parasitic protozoa, and, more recently, opportunistic fungi [2].

In practice, an infectious agent is first genetically modified to stably express a luciferase gene (*LUC*) as a reporter (Figure 1A). Luciferase-expressing cells are then injected into animals and the substrate, luciferin or coelenterazine, is extemporaneously administered (Figure 1B). For bacterial bioreporters, substrate application is not required since prokaryotic *lux* operons include genes encoding luciferase and enzymes for substrate synthesis (long-chain aliphatic aldehyde). However, the bacterial luciferase dependence on FMNH_2_ as a co-substrate makes it less suitable for eukaryotic cells. Finally, regardless of the luciferase system used, light emitted from reporter cells is externally monitored in real time, using sensitive photon detectors based on cooled or intensified charge coupled devices (CCD) [3]. Thus, the special charm of the system results from repeated monitoring of individual animals at different time points.

## What Were (Are) the Hurdles to Overcome in the Development of Fungal Infection BLI Models?

The development of BLI strategies for monitoring infections requires the construction of luciferase-expressing microorganisms that fit the special needs of subsequent experiments. To date, three different luciferase genes have been applied to infection studies on yeast and mold: (i) the firefly (*Photinus pyralis*) *fLUC* gene encoding a luciferase (Fluc), which converts the substrate luciferin to oxy-luciferin in an ATP-dependent manner, and (ii) the sea pansy (*Renilla reniformis*) *rLUC* and (iii) the copepod *Gaussia* (*Gaussia princeps*) *gLUC*, which both encode coelenterazine dependent luciferases (Rluc and Gluc, respectively) (Figure 1A) [2].

For a long time, the use of luciferase reporters in pathogenic fungi has been restricted to gene regulation studies [4]. However, recent technological advances aimed at developing BLI for fungal infections gave a new lease of life to these brilliant reporters. Indeed, a number of shackles have been released that were holding us back from obtaining robust fungal bioreporters (Figure 1C). For instance, efforts have been made to select strong promoters that provide high luciferase expression levels. Moreover, synthetic optimized luciferase sequences have improved light emission from bioluminescent strains (Figure 1C). In particular, the natural subcellular localizations of the Fluc, Gluc and Rluc proteins constituted real hurdles in pioneering works. The last three amino acids, SKL, of the wild-type firefly luciferase correspond to a strong type 1 peroxisomal targeting sequence (PTS1) (Figure 1A). It is now well established that this peroxisomal localization limits the access to the substrate luciferin, resulting in low light emission. As a consequence, PTS1 was removed from the firefly luciferase to design some of the currently available fungal infection BLI models (Figure 1C) [5]. Additionally, species-specific codon-optimization significantly increases luciferase translation efficiency (Figure 1C). Thus, synthetic optimization not only improves reporter strains from filamentous fungi, but appears to be of special importance to *Candida albicans*, which adopted an unconventional genetic code [6]–[10].

Recently, a breakthrough was also achieved via the rational optimization of some *Gaussia* luciferase-based BLI models. Basically, the *Gaussia* luciferase catalyzes an ATP-independent light emission and is naturally secreted from eukaryotic cells [11]. While uptake of the substrate coelenterazine by *C. albicans* cells is limited [7], its uncontrolled secretion may lead to increased background signals, reducing the sensitivity of the system as shown through in vivo monitoring of T cell recruitment in the murine setting [12]. The addition of a glycosylphosphatidylinositol (GPI) (Figure 1C) anchor to the *Gaussia* luciferase allowed a cell surface exposure of the enzyme that potentiates access to the substrate coelenterazine and promotes a focalization of strong photonic emissions from infected sites. This advance now represents the spearhead of powerful *Gaussia* luciferase-based BLI currently available for fungal infections [7], [8].

## What Are the Currently Available BLI Systems for Pathogenic Fungi?

During the last decade, many efforts have been made to develop pioneering models of BLI for the most common opportunistic fungal infections such as candidiasis and aspergillosis.

The first BLI model of fungal infection was obtained for vulvo-vaginal candidiasis using a *C. albicans* strain that constitutively expressed a codon-modified version of the firefly luciferase as a bioreporter [13]. This system was sufficiently sensitive enough to detect bioluminescent *C. albicans* in the vaginal lumen of infected mice when the substrate luciferin was applied to the genital tract [13]. However, this approach failed to detect deep-seated systemic candidiasis. This failure might be largely attributed to the peroxisomal localization of the expressed luciferase, but restricted luciferin substrate permeability of *C. albicans* hyphae formed during infection has also been proposed. Both problems are now circumvented by using the cell surface–anchored *Gaussia* luciferase described above [7]. Unfortunately, this *Candida* bioluminescent strain does not appear to be a convenient reporter for imaging deep-seated *C. albicans* organ infections, because *Gaussia* luciferase emits light at 480 nm (Figure 1A), which is strongly absorbed by hemoglobin. Thus, this excellent model is mainly restricted to use in BLI of various superficial candidiasis models [14], [15].

However, studies on filamentous fungi have indicated that, in principle, hyphae are not completely impermeable to the firefly substrate luciferin, because pioneering BLI models for aspergillosis have successfully been applied. Initially, an *Aspergillus fumigatus* strain expressing a cytosolic-localized version of the firefly luciferase was used for monitoring of invasive aspergillosis [16] and the sensitivity of this system was significantly enhanced by a complete codon-adaptation of the firefly luciferase gene to the codon usage in *A. fumigatus* [10]. Recently, an *A. fumigatus* bioreporter expressing a cell wall–anchored version of the *Gaussia* luciferase was generated. Targeting of the luciferase into the secretory pathway and cell surface exposure of the enzyme were mediated by the incorporation of two additional secretion signals and a GPI-anchoring peptide in the *Gaussia* luciferase sequence. Similar to *C. albicans*, this BLI system is perfectly suited for sensitive detection of the fungal pathogen during cutaneous aspergillosis development in mice, but also failed in monitoring of deep-seated infections [8].

Finally, latest advances in BLI of deep-seated candidiasis look promising. With the above-mentioned knowledge, a completely synthetic firefly luciferase adapted to the codon usage of highly expressed *C. albicans* genes and lacking the PTS1 has been constructed and used to generate a new *C. albicans* bioreporter. First analyses in a murine infection model for disseminated candidiasis allowed successful monitoring of kidney infections with an excellent correlation between light intensity and fungal burden. Furthermore, cryptic host niches were detected that would have been overseen with conventional techniques (M. Brock, unpublished data, manuscript in revision). Thus, complete adaptation of luciferases to the respective fungal host appears essential for maximum sensitivity of the reporter system.

## What Can Be Monitored Using Fungal Infection BLI Models?

The primary vocation of BLI for pathogenic fungal agents is to offer the possibility to follow with extreme accuracy, in real-time and in a non-invasive manner, the proliferation of microorganisms within intact living animals (Figure 1B).

From an ethical point of view, BLI thus allows a significant reduction in the number of animals required for such investigations (compared to traditional analyses performed post mortem), since multiple imaging of the same animal throughout an experiment can be easily carried out. As described above, the currently available fungal BLI infection models include cutaneous, subcutaneous, vaginal, oropharyngeal, and invasive candidiasis caused by *C. albicans* [7], [13], [17], [18], as well as invasive and cutaneous aspergillosis due to *A. fumigatus* [8], [16]. In addition to the spatial and temporal visualization of the infectious *Candida* or *Aspergillus* reporters, BLI now offers the possibility to study a wide range of other host–pathogen interactions, such as biofilm formation [19], [20] or interactions related to the host immune response [21], [22]. Furthermore, BLI opens a new window in monitoring antifungal drug efficacy in different organs during therapy of candidiasis [7], [13], [23] and aspergillosis [10]. Finally, BLI also represents an unprecedented, powerful approach to assist the development of new vaccines against fungal infections [24].

## What Are the Future Challenges for Fungal Infection BLI?

Although recent advances clearly demonstrate the potential of BLI for monitoring cutaneous, subcutaneous, mucosal, and invasive mycosis, the available systems still suffer from major limitations, which have to be overcome to further expand their field of use.

All luciferases essentially require at least small amounts of oxygen (Figure 1A), which may be withdrawn locally by induced immune response in infected niches [5]. This remains a crucial bottleneck that may only be solved by a yet-undiscovered new class of luciferases that generate light independently of oxidation reactions. In addition, although cell surface–exposed *Gaussia* luciferase offers several advantages (small length, facilitated access to the substrate) compared to the large and ATP-hungry firefly luciferase (Figure 1A), its cognate substrate coelenterazine easily undergoes autoxidation (background signal), has a body site distribution that is strongly dependent on the application route (hidden infected sites), and a light emission at a wavelength (Figure 1A) strongly absorbed by hemoglobin (reduction of the signal) [5]. Thus, *Gaussia* luciferase currently only allows visualization of superficial infections. For these reasons, whereas significant progress was made in recent years to develop pioneering bioluminescent systems to monitor fungal infections, solving the equation concerning the improvement of BLI for deep fungal infections seems difficult and, therefore, represents a strong issue to take up. In this way, red-shifted coelenterazine analogues for *Gaussia* luciferase, as well as red-shifted and thermostable firefly luciferases [25] generated through enzyme redesign (Figure 1C), may enhance sensitivity of BLI, overcoming some of the current drawbacks. At the same time, the adaptation of BLI to other important fungal pathogens (as illustrated recently with *Aspergillus terreus* [9]), such as emerging non-albicans *Candida* species, dimorphic fungi, *Cryptococcus*, *Fusarium*, and zygomycetes, will certainly become a main challenge in the field.
